# Geographic variation in seed traits within and among forty-two species of *Rhododendron* (Ericaceae) on the Tibetan plateau: relationships with altitude, habitat, plant height, and phylogeny

**DOI:** 10.1002/ece3.1067

**Published:** 2014-04-19

**Authors:** Yongji Wang, Jianjian Wang, Liming Lai, Lianhe Jiang, Ping Zhuang, Lehua Zhang, Yuanrun Zheng, Jerry M Baskin, Carol C Baskin

**Affiliations:** 1 Key Laboratory of Resource Plants, Beijing Botanical Garden, West China Subalpine Botanical Garden, Institute of Botany, Chinese Academy of SciencesBeijing, Xiangshan, China; 2 University of Chinese Academy of SciencesBeijing, 100039, China; 3 Lushan Botanical Garden, Jiangxi Province and Chinese Academy of SciencesJiujiang, Jiangxi Province, 332900, China; 4 Department of Biology, University of KentuckyLexington, Kentucky, 40506; 5 Department of Plant and Soil Sciences, University of KentuckyLexington, Kentucky, 40546

**Keywords:** Altitude, geographic variation, habitat, plant height, *Rhododendron*, seed mass, seed morphology

## Abstract

Seed mass and morphology are plant life history traits that influence seed dispersal ability, seeding establishment success, and population distribution pattern. Southeastern Tibet is a diversity center for *Rhododendron* species, which are distributed from a few hundred meters to 5500 m above sea level. We examined intra- and interspecific variation in seed mass and morphology in relation to altitude, habitat, plant height, and phylogeny. Seed mass decreased significantly with the increasing altitude and increased significantly with increasing plant height among populations of the same species. Seed mass differed significantly among species and subsections, but not among sections and subgenera. Seed length, width, surface area, and wing length were significantly negative correlated with altitude and significantly positive correlated with plant height. Further, these traits differed significantly among habitats and varied among species and subsection, but not among sections and subgenera. Species at low elevation had larger seeds with larger wings, and seeds became smaller and the wings of seeds tended to be smaller with the increasing altitude. Morphology of the seed varied from flat round to long cylindrical with increasing altitude. We suggest that seed mass and morphology have evolved as a result of both long-term adaptation and constraints of the taxonomic group over their long evolutionary history.

## Introduction

The seed is the most important stage in the life cycle of plants (Baskin and Baskin 2001), and seed traits, including mass, dormancy and dispersal, are central components of plant life histories (Thompson 1987), whose importance to plant fitness is widely appreciated (Moles et al. 2005; Moles et al. 2007; Bolmgren and Cowan 2008; Hallett et al. 2011; Turnbull et al. 2012). Traditionally, seed mass within species was considered to be a remarkably constant characteristic (Bu et al. 2007). However, if resources are limited, a plant may allocate them into many, smaller seeds or into fewer, larger seeds (Moles and Westoby 2003; Pluess et al. 2005; Bu et al. 2007; Guo et al. 2010; Wu et al. 2011). Therefore, seed mass within a species or even an individual plant can vary significantly (Hendrix 1984; Martijena and Bullock 1997; Hodkinson et al. 1998; Guo et al. 2010; Turnbull et al. 2012). Seed mass can vary over 10 orders of magnitude among plant species, and even within a plant community (Leishman and Westoby 1994). Such variation in seed mass often is effected by environmental factors. Both within and among species, a smaller seed mass has been associated with more disturbed habitats, an increase in altitude (Bu et al. 2007) and with an increase in latitude (Pluess et al. 2005).

Numerous recent studies have found it reasonable to expect that seed traits within a species, such as seed mass, could be affected by phylogenetic constraints and developmental allometries (Lord et al. 1995; Tautenhahn et al. 2008; Queenborough et al. 2009; Munzbergova and Plackova 2010; Turnbull et al. 2012). Usually, the variation in seed mass mainly is among seeds within genera or even families (Wolfe 1995); however, differences in seed mass of the same plant are small (Mazer 1990; Lord et al. 1995). The reason for low variation in seed mass is phylogenetic constraints or niche conservatism (Lord et al. 1995). Adaptive changes may be restricted by species' evolutionary history, that is, complex patterns of covariation among functionally related traits (Baker 1972; Pigliucci 2003; Pluess et al. 2005). Generally, variation in seed traits and its causes is unclear (Bu et al. 2007).

**Figure 1:**
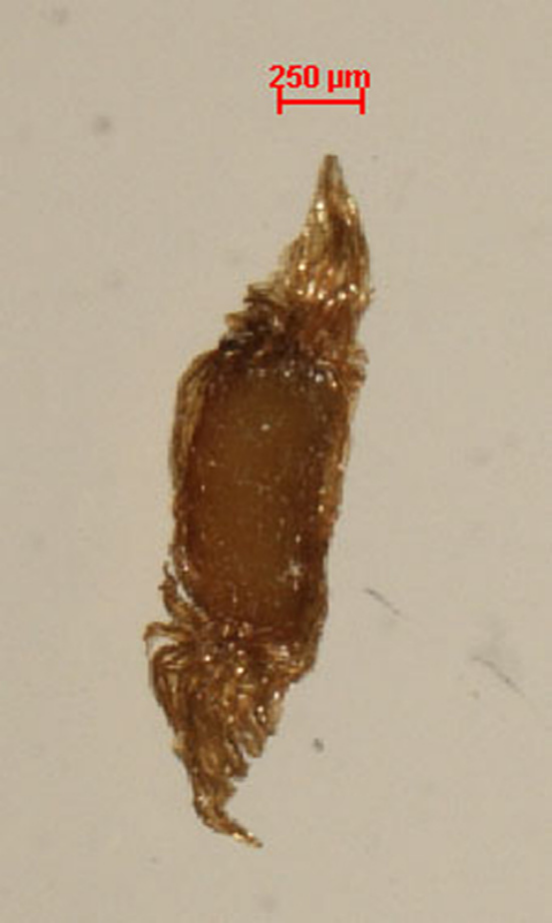
Seed of *Rhododendron delavayi var. peramoenum*.

We examined the relationship between elevation, plant height, habitat, phylogeny, and seed traits among 59 populations representing 42 species of *Rhododendron* in the southeast Tibetan Plateau. Further, we selected three species that occur over a wide altitudinal gradient and have large differences in seed mass and seed dispersal capacities (*R*. *thomsonii R*. *cerasinum*, and *R*. *aganniphum* var. *flavorufum*) to investigate variation in seed traits along the elevation gradients. In general, ripe seeds of *Rhododendron* are oval, flat, and reddish brown, but they vary with environment (Fig. 1).

We hypothesized that the seeds of *Rhododendron* species would vary greatly in seed traits as a result of their adaptation to extremely different environments. Specifically, we addressed the following question: Are seed mass and morphology related to and vary with elevation, plant height, and phylogeny? It is necessary to answer this question in order to understand how plants are adapted the extreme and unique environments in Himalaya.

## Materials and Methods

### Study sites and sampling methods

*Rhododendron* (Ericaceae) is one of the largest genera of angiosperms, and it includes nine subgenera and more than 1000 species. The genus is widely distributed in Asia, Europe, and north America (Fang et al. 2005), ranging from 65° north to 20° south latitude in the tropical, temperate, and boreal zones. Altitudinally, it occurs in vegetation zones that range from a few hundred meters to about 5500 m above sea level, including subtropical mountain evergreen broad-leaved forest, coniferous and mixed broad-leaved forest, coniferous forest, the open-like coniferous forest, elfin forest, and *Rhododendron* shrub. The morphology of *Rhododendron* plants and seeds varies significantly across this environmental complex.

The Himalaya is the highest mountain chain in the world and has a complex of ecological environments. From low to high elevation, the vegetation consists of four types: tropical, subtropical, temperate, and alpine. The Himalayan region is the distribution center of *Rhododendron* (Fang and Lu 1981), and in China, there are 351 species, including three subgenera, six sections, and 41 subsections containing 36% of the species in the genus. In addition, the region also is the diversification center of the genus, where it is taxonomically very complex (Fang & Lu 1981). In this region, *Rhododendron* species grow over an altitudinal range from a few hundred meters to about 5500 m, which is an ideal situation for studying variation in size and morphology of seeds.

**Figure 2:**
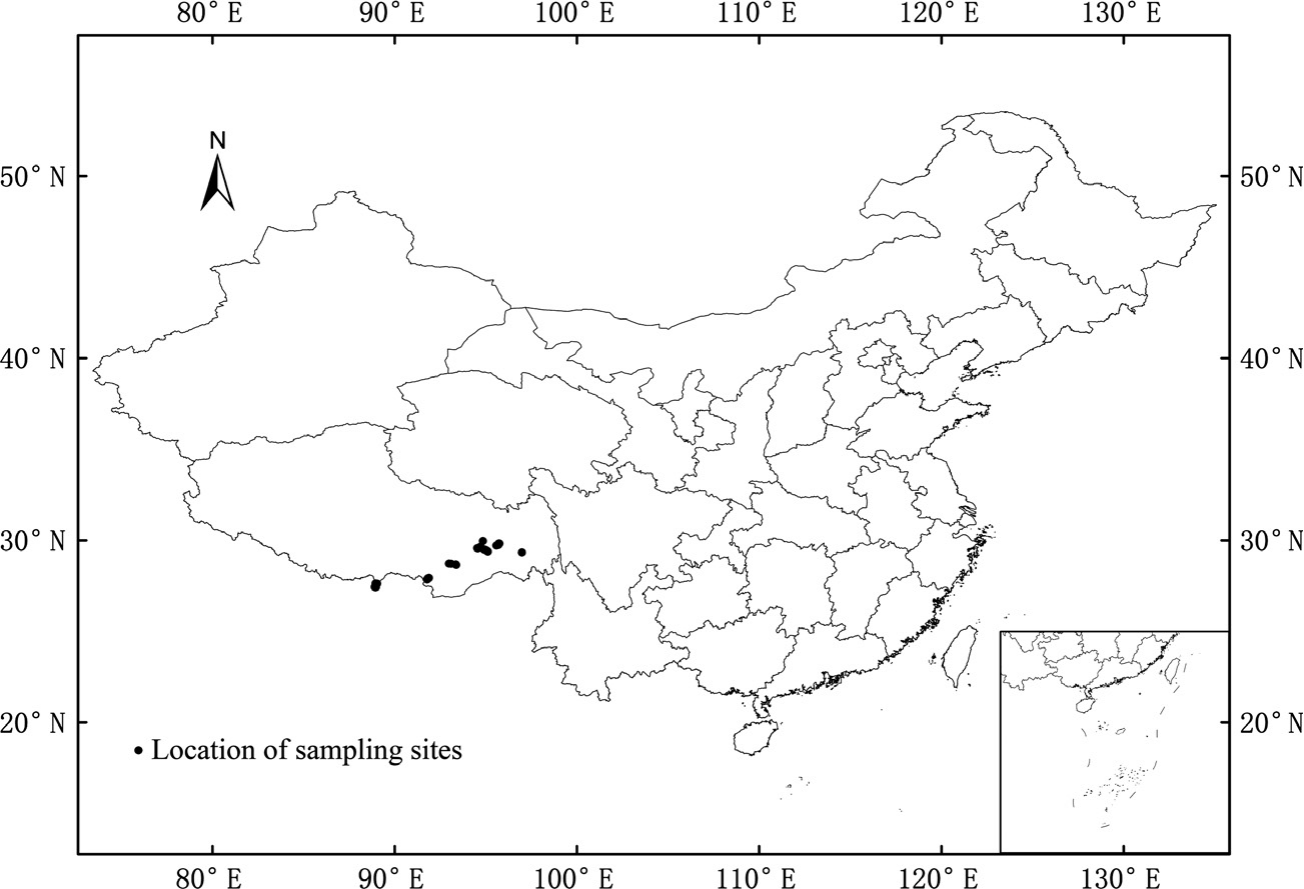
Map of China showing location of sampling sites on the Tibetan Plateau.

The study sites are located on the southeastern Tibet plateau (27.239°–29.996°, 88.5–97.287°) near the Himalayan and Hengduan Mountains (Fang et al. 2005). The altitude ranges from 2280 to 4540 m, and the region includes Milin, Motuo, Bomi, Cuona, Longzi, Yadong, Linzhi, and Chayu counties (Fig. 2). In 2010, we investigated the seeds of 42 *Rhododendron* species (59 populations) in three subgenera, three sections, and 23 subsections (Table 1). Habitats of the sampling sites included alpine shrub (AS), rocky slope (RS), and forest (F).

**Table 1:** Environment variables and seed mass (Mean ± SD, *n* = 3, 1000 seeds for each replicate) of 59 populations of 42 congeneric *Rhododendron* species

Population	Species	Altitude	Habitat	Mean height of plant (m)	Subgenus	Section	Subsection	Seed mass (g)
1	*Rhododendron vellereum*	4220	1	2	1	1	10	0.0713 ± 0.0006
2	*Rhododendron tsariense*	4170	1	1.3	1	1	22	0.0550 ± 0.0002
3	*Rhododendron faucium*	3940	1	0.5	1	1	13	0.1275 ± 0.0024
4	*Rhododendron faucium*	3830	3	1.5	1	1	13	0.0684 ± 0.0032
5	*Rhododendron faucium*	3570	3	2.5	1	1	13	0.0658 ± 0.0008
6	*Rhododendron faucium*	4200	3	1.3	1	1	13	0.0748 ± 0.0013
7	*Rhododendron faucium*	3220	2	1.6	1	1	13	0.0775 ± 0.0023
8	*Rhododendron catacosmum*	4130	3	3	1	1	2	0.1161 ± 0.0005
9	*Rhododendron calvescens*	3600	3	4	1	1	6	0.1491 ± 0.0031
10	*Rhododendron principis*	3760	3	3.5	1	1	10	0.0772 ± 0.0017
11	*Rhododendron trichocladum*	3660	3	1	3			0.0757 ± 0.0006
12	*Rhododendron hookeri*	3680	2	1	1	1	13	0.0829 ± 0.0017
13	*Rhododendron megalanthum*	3210	2	2.5	1	1	13	0.0536 ± 0.0019
14	*Rhododendron megalanthum*	2600	2	2	1	1	13	0.0560 ± 0.0066
15	*Rhododendron maddenii* subsp*. Crassum*	2650	2	2	2	2	14	0.1257 ± 0.0031
16	*Rhododendron maddenii* subsp. *Crassum*	2670	3	2.5	2	2	14	0.1499 ± 0.0008
17	*Rhododendron arboreum* var. *roseum*	2510	3	4	1	1	17	0.0207 ± 0.0047
18	*Rhododendron setiferum)*	3570	3	3	1	1	6	0.0940 ± 0.0005
19	*Rhododendron coriaceum*	3260	3	3	1	1	12	0.0543 ± 0.0021
20	*Rhododendron kongboense*	4450	1	0.1	2	3		0.0817 ± 0.0009
21	*Rhododendron keysii*	2900	3	4	1	1	8	0.0703 ± 0.0007
22	*Rhododendron lacteum*	4040	2	1	1	1	10	0.0918 ± 0.0096
23	*Rhododendron lacteum*	4540	1	0.4	1	1	10	0.1152 ± 0.0005
24	*Rhododendron lacteum*	4000	3	1.5	1	1	10	0.0670 ± 0.0034
25	*Rhododendron grande*	2760	2	3	1	1	1	0.1764 ± 0.0106
26	*Rhododendron heliolepis*	3420	3	1.5	2	2	16	0.0522 ± 0.0017
27	*Rhododendron heliolepis*	3570	3	1	2	2	21	0.0858 ± 0.0027
28	*Rhododendron lulangense*	3170	2	1.5	1	1	10	0.1222 ± 0.0006
29	*Rhododendron bainbridgeanum*	4000	1	1	1	1	6	0.0764 ± 0.0006
30	*Rhododendron trichostomum*	4490	1	0.3	2	3		0.0828 ± 0.0014
31	*Rhododendron agastum*	3570	3	1.7	1	1	7	0.0685 ± 0.0011
32	*Rhododendron erosum*	3140	3	4	1	1	5	0.0993 ± 0.0062
33	*Rhododendron triflorum*	3150	3	2	2	2	21	0.2845 ± 0.0048
34	*Rhododendron arboreum*	3140	3	6	1	1	17	0.0718 ± 0.0001
35	*Rhododendron pruniflorum*	4170	1	1.1	1	1	20	0.0263 ± 0.0015
36	*Rhododendron sinogrande*	2640	2	5	1	1	1	0.1980 ± 0.0224
37	*Rhododendron pendulum*	2870	3	1.2	2	2	15	0.0607 ± 0.0007
38	*Rhododendron mekongense*	3940	1	0.4	3			0.0418 ± 0.0011
39	*Rhododendron mekongense*	3690	1	1.5	3			0.0793 ± 0.0013
40	*Rhododendron campylogynum*	4350	1	0.1	2	2	23	0.0193 ± 0.0009
41	*Unnamed species*	3690	1	2.8	1	1		0.0726 ± 0.0005
42	*Rhododendron delavayi var. peramoenum*	2280	2	2.5	1	1	17	0.0758 ± 0.0009
43	*Rhododendron erythrocalyx*	3490	3	1.5	1	1	6	0.0755 ± 0.0001
44	*Rhododendron calostrotum var. calciphilum*	4150	1	0.2	2	2	19	0.0235 ± 0.0006
45	*Rhododendron kyawi*	3210	2	3	1	1	4	0.1069 ± 0.0017
46	*Rhododendron kyawi*	2920	3	3.5	1	1	4	0.0978 ± 0.0055
47	*Rhododendron aperantum*	4010	1	0.3	1	1	2	0.0393 ± 0.0008
48	*Rhododendron nivale*	4450	1	0.1	2	2	9	0.1046 ± 0.0005
49	*Rhododendron aganniphum*	4530	1	1.4	1	1	10	0.0689 ± 0.0003
50	*Rhododendron aganniphum*	4170	1	1	1	1	10	0.1201 ± 0.0500
51	*Rhododendron stewartianum*	3720	2	1.2	1	1	13	0.0666 ± 0.0020
52	*Rhododendron stewartianum*	2900	3	2.5	1	1	13	0.0422 ± 0.0015
53	*Rhododendron stewartianum*	3210	3	1	1	1	13	0.0710 ± 0.0023
54	*Rhododendron stewartianum*	3260	3	2.5	1	1	13	0.0887 ± 0.0001
55	*Rhododendron hirtipes*	4130	3	2	1	1	6	0.1002 ± 0.0008
56	*Rhododendron campanulatum*	4040	1	2	1	1	3	0.0972 ± 0.0016
57	*Rhododendron campanulatum*	3570	3	2	1	1	3	0.1060 ± 0.0006
58	*Rhododendron uvarifolium*	3150	3	2	1	1	18	0.0967 ± 0.0058
59	*Rhododendron uvarifolium*	3600	3	3	1	1	18	0.1182 ± 0.0009

Habitat: 1, alpine shrub; 2, rocky slope; 3, forest.

Subgenus: 1, Subgen. *Hymenanthes* (Blume) K. Koch; 2, Subgen. *Rhododendron*; 3, Subgen. *Pseudazalea* Sleumer.

Section c: 1, Sect. *Ponticum* G. Don; 2, Sect. *Rhododendron*; 3, Sect. *Pogonanthum* G. Don.

Subsection: 1, subsect. *Grandia* Sleumer; 2, subsect. *Neriiflora* Sleumer; 3, subsect. *Campanulata* Sleumer; 4, subsect. *Parishia* Sleumer; 5, subsect. *Barbata* Sleumer; 6, subsect. *Selensia* Sleumer; 7, subsect. *Irrorata* Sleumer; 8, subsect. *Cinnabarina* (Hutch.) Sleumer; 9, subsect. *Lapponica* (Balf. F.) Sleumer; 10, subsect. *Taliensia* Sleumer; 11, subsect. *Barbata* Sleumer; 12, subsect. *Falconera* Sleumer; 13, subsect. *Thomsonii* Sleumer; 14, subsect. *Maddenia* (Hutch.) Sleumer; 15, subsect. *Edgeworthia* (Hutch.) Sleumer; 16, subsect. *Heliolepida* (Hutch.) Sleumer; 17, subsect. *Arborea* Sleumer; 18, subsect. *Fulva* Sleumer; 19, subsect. *Saluenensia* (Hutch.) Sleumer; 20, subsect. *Glauca* (Hutch.) Sleumer; 21, subsect. *Triflora* (Hutch.) Sleumer; 22, subsect. *Lanata* Chamb; 23, subsect. *Campylogyna* (Hutch.) Sleumer.

Seeds were collected by hand from more than ten individual plants randomly selected from three to five subpopulations at each altitude in late September and early October 2010. Seeds of each species and subpopulation were pooled, and mean seed mass of each species within a population at each altitude was determined. Three to five mature but unopened fruits were collected from each infructescence on a plant. To reduce variation among individuals due to potential effects of fruit position on seed mass, we collected fruits at basal, middle, and distal positions on each sampled infructescence. These fruits were dissected, and the seeds were removed and air-dried until used. Seeds were divided into batches of 1000 air-dried under ambient laboratory condition seeds, and three batches per population site were weighed to the nearest 0.0001 g on an electronic balance.

Plant height of each sampled individual was measured to the nearest decimeter. Seed morphology, including seed length, seed width, seed thickness, seed wing length at hilium, seed wing length at chalaza, and seed wing length on lateral sides, was measured for three replicates of 30 seeds each.

To assess variation in seed traits along the altitudinal gradient for a single species, we selected three species that differ in seed size and grow in different alpine habitats, but with a similar distribution over a large altitudinal gradient. The altitudinal gradient extended ca. 980 m for *R. thomsonii*, 540 m in *R. aganniphum var. flavorufum,* and 868 m for *R. cerasinum* (Table 2). Seed wing length and seed surface area were calculated using the following equations:

**Table 2:** The GLM results of the relationship between seed traits and altitude, plant height, habitat, subgenus, section, subsection, and species

Variables		Source						
	
		Altitude	Plant height	Habitat	Subgenus	Section	Subsection	Species
Seed mass	df	3	4	2	2	2	21	41
	F	11.744	3.681	1.909	0.672	0.42	2.701	5.29
	Sig.	**<0.01**	**<0.01**	**<0.05**	0.515	0.659	**<0.01**	**<0.01**
	*R*^2^	0.06	0.032	0.242	0.005	0.003	0.133	0.184
Seed length	df	3	4	2	1	2	14	36
	F	2.102	1.335	6.839	0.989	5.223	1.945	3.372
	Sig.	**<0.05**	0.271	**<0.01**	0.326	**<0.05**	**<0.05**	**<0.01**
	*R*^2^	0.028	0.003	0.01	0.001	0.005	0.01	0.039
Seed width	df	3	4	2	1	2	14	36
	F	1.801	1.638	4.341	0.093	0.672	3.218	9.117
	Sig.	**<0.05**	0.18	**<0.05**	0.762	0.517	**<0.05**	**<0.01**
	*R*^2^	0.004	0.005	0.007	0	0.001	0.011	0.041
Seed thickness	df	3	4	2	1	2	14	36
	F	0.175	1.677	1.301	0.264	0.144	1.587	0.442
	Sig.	0.548	1.171	0.281	0.61	0.866	0.158	0.977
	*R*^2^	0.001	0.003	0.017	0.002	0.002	0.188	0.515
Seed length to width	df	3	4	2	1	2	14	36
	F	0.116	0.994	0.45	1.952	4.337	1.469	1.157
	Sig.	0.951	0.42	0.64	0.17	**<0.05**	0.2	0.394
	*R*^2^	0.0001	0.0001	0	0.001	0.003	0.007	0.011
Seed width to thickness	df	3	4	2	2	2	17	36
	F	3.798	3.295	4.908	6857	2.624	2.663	2.827
	Sig.	0.16	**<0.05**	**<0.01**	**<0.01**	0.083	**<0.05**	**<0.05**
	*R*^2^	0.003	0.004	0.167	0.219	0.102	0.618	0.872
Seed surface area	df	3	4	2	1	2	14	36
	F	2.358	1.556	6.648	0.533	2.419	3.243	6.14
	Sig.	0.083	0.202	**<0.01**	0.47	0.103	**<0.01**	**<0.01**
	*R*^2^	0.128	0.114	0.029	0.001	0.007	0.034	0.127
Seed wing length	df	3	4	2	1	2	14	33
	F	6.376	2.962	5.632	2.658	14.87	5.417	5.857
	Sig.	**<0.01**	**<0.05**	**<0.01**	0.111	**<0.01**	**<0.01**	**<0.01**
	*R*^2^	0.03	0.021	0.03	0.008	0.055	0.082	0.941

Note: Analysis of variance in bold type is statistically significant at *P* < 0.05.


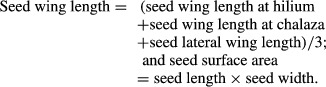


### Data analysis

The relationship between altitude and seed traits for every population was determined with a parametric Pearson's product moment (*r*) test. An ANOVA analysis was used to test the effects of habitat, subgenus, section, subsection, and species. When a dataset was unbalanced or included categorical variables, GLM was used for variance analysis. Coefficient of variation (CV) of seed mass was calculated as standard deviation of seed mass (SD) × 100/mean seed mass (Pluess et al. 2005). All statistics analyses were performed with the Statistical Package for the Social Sciences version 18.0 (SPSS, Inc., Chicago, IL).

## Results

### Variation in seed traits among populations

**Figure 3:**
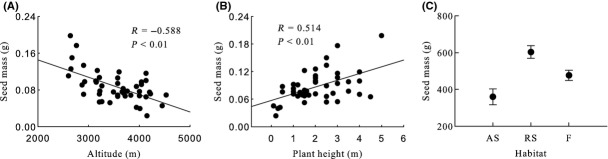
Variation in seed mass with altitude, plant height, and habitat. (A) Correlation between seed mass and altitude; (B) Correlation between seed mass and plant height; (C) variation in seed mass in alpine shrub (AS), rocky slope (RS), and forest (F).

**Figure 4:**
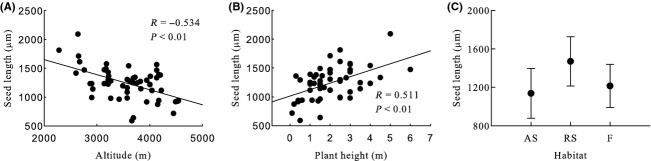
Variation in seed length with altitude, plant height, and habitat. (A) Correlation between seed length and altitude; (B) Correlation between seed length and plant height; and (C) Variation in seed length in alpine shrub (AS), rocky slope (RS), and forest (F).

**Figure 5:**
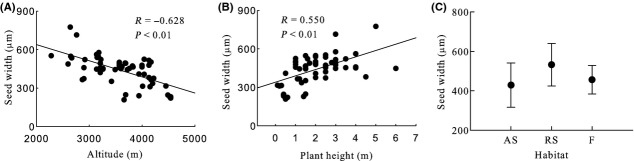
Variation in seed width with altitude, plant height, and habitat. (A) Correlation between seed width and altitude; (B) Correlation between seed width and plant height; and (C) variation in seed width in alpine shrub (AS), rocky slope (RS), and forest (F).

**Figure 6:**
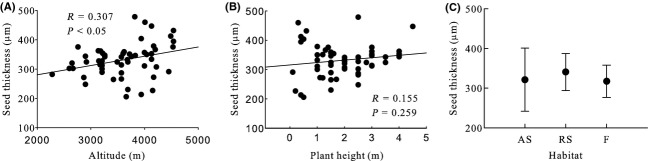
Variation in seed thickness with altitude, plant height, and habitat. (A) Correlation between seed thickness and altitude; (B) Correlation between seed thickness and plant height; and (C) Variation in seed thickness in alpine shrub (AS), rocky slope (RS), and forest (F).

**Figure 7:**
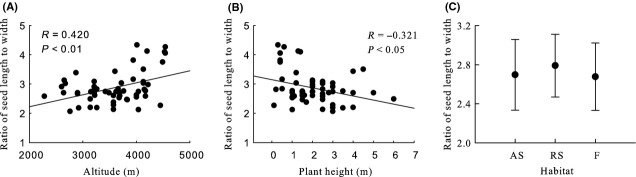
Variation in ratio of seed length to width with altitude, plant height, and habitat. (A) Correlation between ratio of seed length to width and altitude; (B) Correlation between the ratio of seed length to width and plant height; and (C) Variation in ratio of seed length to width in alpine shrub (AS), rocky slope (RS), and forest (F).

**Figure 8:**
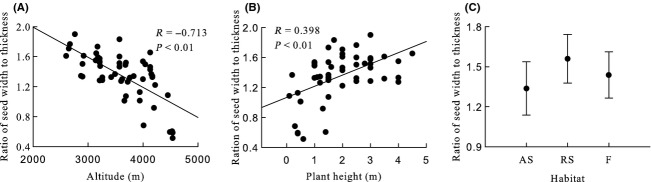
Variation in ratio of seed width to thickness with altitude, plant height, and habitat. (A) Correlation between ratio of seed width to thickness and altitude; (B) Correlation between ratio of seed width to thickness and plant height; and (C) Variation in ratio of seed width to thickness in alpine shrub (AS), rocky slope (RS), and forest (F).

**Figure 9:**
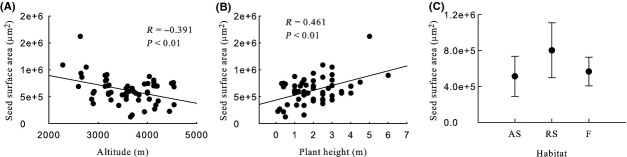
Variation in seed surface area with altitude, plant height, and habitat. (A) Correlation between seed surface area and altitude; (B) Correlation between seed surface area and plant height; and (C) Variation in seed surface area in alpine shrub (AS), rocky slope (RS), and forest (F).

**Figure 10:**
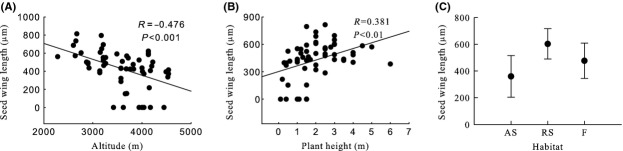
Variation in seed wing length with altitude, plant height, and habitat. (A) Correlation between seed wing length and altitude; (B) Correlation between seed wing length and plant height; and (C) Variation in seed wing length in alpine shrub (AS), rocky slope (RS), and forest (F).

Generally, seed mass, seed length, seed width, ratio of seed width to thickness, seed surface area, and seed wing length were significantly negative correlated with altitude, and seed thickness and ratio of seed length to width were significantly positive correlated with altitude (Table 2, Figs 3A, 4A, 5A, 6A, 7A, 8A, 9A, and 10A).

Seed mass, seed length, seed width, ratio of seed width to thickness, seed surface area, and seed wing length were significantly positive correlated with plant height, and ratio of seed length to width was significantly negative correlated with plant height. Seed thickness was not significantly correlated with plant height (*R* = 0.155, *P* = 0.259) (Figs 3B, 4B, 5B, 6B, 7B, 8B, 9B, and 10B).

Habitat, subgenera, section, subsection, and species had various effects on seed traits.

#### Seed mass

Habitat, subsection, and species had significant effects on seed mass, but subgenera and section did not (Table 2). Seed mass was highest for rocky slope and lowest for alpine shrub (Table 2, Fig. 3C).

#### Seed length

Habitat, section, and species had significant effects on seed length, but subgenera and subsections did not (Table 2). Seed length was highest for rocky slope and lowest for alpine shrub (Table 2, Fig. 4C).

#### Seed width

Habitat, subsection, and species had significant effects on seed width (Table 2). Seed width was highest for rocky slope and lowest for alpine shrub (Table 2, Fig. 5C).

#### Seed thickness, ratio of seed length to width, and ratio of seed width to thickness

None of these three seed traits differed significantly among habitats, subgenera, sections, subsections, or species (Table 2, Fig. 6C, 7C, and 8C).

#### Seed surface area

Seed surface area differed significantly among habitats, subsections, and species, but not among subgenera or sections (Table 2). Seed surface area was highest for rocky slope (Table 2, Fig. 9C).

#### Seed wing length

Seed wing length differed significantly among habitats, sections, subsections, and species (Table 2). Seed wing length was highest for rock slope and lowest for alpine shrub (Table 2, Fig. 10C).

### Variation in seed traits among populations of same species

**Figure 11:**
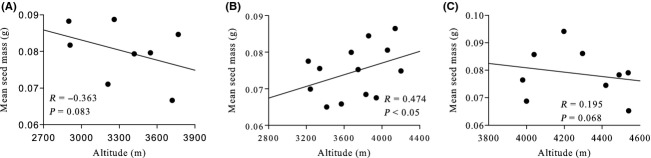
Relationships between seed mass and altitude among populations of (A) *R. thomsonii* (B) *R. aganniphum var. flavorufum* (C) *R. cerasinum*.

Seed mass was significantly correlated with altitude in *R. aganniphum var. flavorufum* (*R* = 0.474, *P* < 0.05, Fig. 11B) but not in *R. thomsonii* (*R* = −0.363, *P* = 0.083, Fig. 11A) or *R. cerasinum* (*R* = 0.195, *P* = 0.068 Fig. 11C).

Variation in seed mass among populations was relatively high, with CVs of 23.1% for *R. aganniphum* var. *flavorufum*, 21.3% for *R. thomsonii,* and 15.44% for *R. cerasinum* (Table 3).

**Table 3:** Variation in seed mass (Mean ± SD, *n* = 3, 1000 seeds for each replicate) of three *Rhododendron* species

Species	Number of populations	Altitudinal range (m)	Thousand seed weight (g) (mean ± SD)	CVs among populations (%)
*Rhododendron thomsonii*	13	3220–4200	0.0792 ± 0.0169	21.3
*Rhododendron aganniphum* var. *flavorufum*	8	4000–4540	0.0932 ± 0.0142	23.1
*Rhododendron cerasinum*	8	2900–3768	0.0704 ± 0.0161	15.44

The effect of plant height and habitat on other seed traits was not significant among populations within a single species.

## Discussion

### Seed trait responses to altitude

#### Seed mass variation among congeneric species

We detected a significant negative correlation between seed mass and altitude among the 59 *Rhododendron* populations and a negative correlation between elevation and seed mass among populations of the same species. These results were in accordance with two previous studies (Baraloto et al. 2005). A negative relationship also was found between altitude and seed mass in a flora of the eastern Tibetan Plateau (Bu et al. 2007). A decrease in seed mass with altitude may be due to plastic responses induced by the environment caused by a decline in resource availability. Low temperatures at higher altitudes may reduce photosynthetic rates, and a shorter growing seasons may reduce the time for seed development and seed provisioning, thereby reducing mature seed mass (Baker 1972). The smaller seeds at high altitude also may evolve by natural selection if the growing season is not long enough to produce large seeds (Venable and Rees 2008).

In contrast, Pluess et al. (2005) reported a positive correlation between seed mass and altitude across species and within species. Pluess et al. (2005) argued that natural selection should favor production of larger seeds in species at higher altitudes because larger seeds exhibit superior survivorship in stressful environments, which accounted for the pattern they observed. The ecological sorting of species across elevations could also generate this pattern directly on seed mass. Indeed, the fact that Pluess et al. (2005) found no relationship between seed mass and altitude within species argues against a strong role for in situ natural selection. Therefore, although seed mass has been found to be associated with altitude, the patterns observed are not highly consistent, and the underlying mechanisms have not been identified.

#### Seed morphology variation among congeneric species

In our study, seed morphology varied with environmental factors. The ratio of seed length to width and seed width for *Rhododendron* species were positively correlated with altitude. Seed length, seed width, ratio of seed width to thickness, seed surface area, and seed wing length had a significant negative relationship with the increasing altitude. Seed morphology was related to seed dispersal (Howe and Smallwood 1982). Seeds of *Rhododendron* species mainly are dispersed by wind, but wind speed is relatively low in the low-altitude area. However, the flat, circular shape maximizes surface area, and the large wings are conductive to flight. With the increasing altitude, the wind becomes stronger and thus more conducive for seed dispersal. Seed and seed wing are narrow at high altitudes, which reduces flight ability. The possible explanation of this pattern is that the wind at high altitudes is strong enough for seed dispersal, and seeds do not need to develop significant structures for flight.

### Correlation between seed trait and plant height

#### Seed mass variation

We detected a significant positive relationship between seed mass and plant height (Fig. 5). Some studies have reported a positive relationship because the data sets included species representing many growth forms (Moles and Westoby 2004; Grubb et al. 2005; Venable and Rees 2008; Queenborough et al. 2009). For example, herbs produce relatively smaller seeds than woody plants (Mazer and Percival 1989; Leishman et al. 1995). In our study, all species are woody; thus, variation due to growth form is avoided. Seed mass was significantly positively correlated with plant height among populations across species, but not within species, which is in accordance with the study by Moles and Westoby (2004). This suggests that mechanisms are different at different taxonomical levels. Positive correlations are more often found among a taxonomically highly diverse group of taxa, and the reason may be phylogenetic constraints.

Increase in seed mass with plant height has been proposed to reflect adaptive responses to dispersal requirements and to architectural constraints or competitive interactions among seedlings (Grubb et al. 2005; Moles et al. 2007). Alternatively, differences in seed mass and plant height among populations or taxa may be due to plastic responses to local environmental conditions (Baker 1972; Moles et al. 2005; Guo et al. 2010).

#### Variation in seed morphology

Species with high dispersal ability may be more widely distributed than those with low dispersal ability (Gutierrez and Menéndez 1997). Dispersal ability is significantly correlated with the seed mass (Rees 1995). Smaller and lighter seeds are readily transported by dispersal agents (Venable and Brown 1988; Greene and Johnson 1993), and thus they have an advantage in colonization and in becoming abundant. Larger and heavier seeds are relatively less abundant, but they can produce seedlings that are more competitive than those produced by small seeds, which enable them to establish and survive under various stress conditions such as defoliation, shading, competition, herbivory, drought, and disturbance (Armstrong & Westoby 1993). We found that taller plants of *Rhododendron* had larger seeds and seed wings compared to shorter plants. With decrease in plant height, the seed wing became smaller and even disappeared, presumably because there is not enough energy to be allocated to production of wings. There is a trade-off between plant growth and production of wings (Ginwal et al. 2005).

### Seed trait responses to habitat

Seed mass, seed length, seed width, ratio of seed width to thickness, seed surface area, and seed wing length varied significantly among habitats for populations of the same species. These traits had their highest values in rocky slope habitat, and the reason may be that, compared with forest and alpine shrub, the plants on rocky slopes are exposed to high solar irradiance. Thus, the plants had more energy for reproductive growth and production of large seeds, which are more favorable for germination and seedling growth.

### Seed trait responses to phylogeny

Mass and morphology of the *Rhododendron* seeds were correlated with taxonomic membership mainly at the species and subsection levels. This phylogenetic pattern of seed size previously has been shown for different kinds of genera (Kelly et al. 1996; Westoby et al. 1996; Hodkinson et al. 2002). However, two corresponding questions remain unsolved: How to interpret this phylogenetic correlation, and how to consider both phylogenetic and ecological correlations.

Seed mass and morphology might be the result of both selective pressure over long-term ecological time and the constraints over long evolutionary history of the taxonomic group. Thus, seed mass will be similar in more closely related species regardless of ecological factors. Therefore, we maintain that correlates of ecology and phylogeny should be taken into account in comparative studies on seed mass and morphology among species.

## Conclusions

In summary, our results indicate elevation, habitat, plant height, and phylogeny were all correlated with seed mass and morphology among species of *Rhododendron*. We found a selection pressure for species with lighter and smaller seeds, and shorter seed wings at higher altitude. Seed mass was in positive correlation with plant height, seed traits varied with habitats, and phylogeny constrains the seed traits variation.

## References

[b100] Armstrong DP, Westoby M (1993). Seedlings from large seeds tolerate defoliation better – a test using phylogenetically independent contrasts. Ecology.

[b3] Baker HG (1972). Seed weight in relation to environmental conditions in California. Ecology.

[b4] Baraloto C, Forget PM, Goldberg DE (2005). Seed mass, seedling size and neotropical tree seedling establishment. J. Ecol.

[b5] Baskin CC, Baskin JM (2001). Seeds: ecology, biogeography, and evolution of dormancy and germination.

[b8] Bolmgren K, Cowan PD (2008). Time - size tradeoffs: a phylogenetic comparative study of flowering time, plant height and seed mass in a north-temperate flora. Oikos.

[b9] Bu H, Chen X, Xu X, Liu K, Jia P, Du G (2007). Seed mass and germination in an alpine meadow on the eastern Tsinghai-Tibet plateau. Plant Ecol.

[b12] Fang M, Fang R, He M, Hu L, Yang H, Chamberlain D (2005). Rhododendron. Flora of China.

[b200] Fang RZ, Min TL (1981). The influence of uplift of Himalayas on the floristic formation of genus *Rhododendron*. Acta Bot. Yunnan.

[b300] Ginwal HS, Phartyal SS, Rawat PS, Srivastava RL (2005). Seed source variation in morphology, germination and seedling growth of *Jatropha curcas* Linn. in central India. Silvae Genet.

[b14] Greene DF, Johnson EA (1993). Seed mass and dispersal capacity in wind-dispersed diaspores. Oikos.

[b15] Grubb PJ, Coomes DA, Metcalfe DJ (2005). Comment on” a brief history of seed size”. Science.

[b16] Guo H, Mazer SJ, Du GZ (2010). Geographic variation in seed mass within and among nine species of *Pedicularis* (Orobanchaceae): effects of elevation, plant height and seed number per fruit. J. Ecol.

[b400] Gutierrez D, Menéndez R (1997). Patterns in the distribution, abundance and body size of carabid beetles (Coleoptera: Caraboidea) in relation to dispersal ability. J. Biogeogr.

[b17] Hallett LM, Standish RJ, Hobbs RJ (2011). Seed mass and summer drought survival in a Mediterranean-climate ecosystem. Plant Ecol.

[b19] Hendrix SD (1984). Variation in seed weight and its effects on germination in Pastinaca sativa L. (Umbelliferae). Am. J. Bot.

[b500] Hodkinson DJ, Askew AP, Thompson K, Hodgson JG, Bakker JP, Bekker RM (1998). Ecological correlates of seed size in the British flora. Funct. Ecol.

[b20] Hodkinson D, Askew A, Thompson K, Hodgson J, Bakker J, Bekker R (2002). Ecological correlates of seed size in the British flora. Funct. Ecol.

[b21] Howe HF, Smallwood J (1982). Ecology of seed dispersal. Annu. Rev. Ecol. Syst.

[b22] Kelly CK, Woodward FI, Crawley M (1996). Ecological correlates of plant range size: taxonomies and phylogenies in the study of plant commonness and rarity in Great Britain [and Discussion]. Philos. Trans. Royal Soc. London Series B: Biol. Sci.

[b26] Leishman MR, Westoby M (1994). Hypotheses on seed size: tests using the semiarid flora of western New South Wales, Australia. Am. Nat.

[b27] Leishman MR, Westoby M, Jurado E (1995). Correlates of seed size variation: a comparison among five temperate floras. J. Ecol.

[b30] Lord J, Westoby M, Leishman M (1995). Seed size and phylogeny in six temperate floras: constraints, niche conservatism, and adaptation. Am. Nat.

[b33] Martijena NE, Bullock SH (1997). Geographic variation in seed mass in the chaparral, shrub *Heteromeles arbutifolia* (Rosaceae). Southwestern Nat.

[b600] Mazer SJ (1990). Seed mass of Indiana dune genera and families – taxonomic and ecological correlates. Evol. Ecol.

[b35] Mazer DB, Percival EF (1989). Ideology or experience? The relationships among perceptions, attitudes, and experiences of sexual harassment in university students. Sex Roles.

[b37] Moles AT, Westoby M (2003). Latitude, seed predation and seed mass. J. Biogeogr.

[b38] Moles AT, Westoby M (2004). Seedling survival and seed size: a synthesis of the literature. J. Ecol.

[b39] Moles AT, Ackerly DD, Webb CO, Tweddle JC, Dickie JB, Westoby M (2005). A brief history of seed size. Science.

[b40] Moles AT, Ackerly DD, Tweddle JC, Dickie JB, Smith R, Leishman MR (2007). Global patterns in seed size. Glob. Ecol. Biogeogr.

[b41] Munzbergova Z, Plackova I (2010). Seed mass and population characteristics interact to determine performance of Scorzonera hispanica under common garden conditions. Flora.

[b44] Pigliucci M (2003). Phenotypic integration: studying the ecology and evolution of complex phenotypes. Ecol. Lett.

[b45] Pluess AR, Schutz W, Stocklin J (2005). Seed weight increases with altitude in the Swiss Alps between related species but not among populations of individual species. Oecologia.

[b46] Queenborough SA, Mazer SJ, Vamosi SM, Garwood NC, Valencia R, Freckleton RP (2009). Seed mass, abundance and breeding system among tropical forest species: do dioecious species exhibit compensatory reproduction or abundances?. J. Ecol.

[b700] Rees M (1995). Community structure in sand dune annuals – is seed weight a key quantity. J. Ecol.

[b50] Tautenhahn S, Heilmeier H, Gotzenberger L, Klotz S, Wirth C, Kuhn I (2008). On the biogeography of seed mass in Germany – distribution patterns and environmental correlates. Ecography.

[b51] Thompson K (1987). Seeds and seed banks. New Phytol.

[b55] Turnbull LA, Philipson CD, Purves DW, Atkinson RL, Cunniff J, Goodenough A (2012). Plant growth rates and seed size: a re-evaluation. Ecology.

[b58] Venable DL, Brown JS (1988). The selective interactions of dispersal, dormancy, and seed size as adaptations for reducing risk in variable environments. Am. Nat.

[b59] Venable DL, Rees M (2008). The scaling of seed size. J. Ecol.

[b61] Westoby M, Leishman M, Lord J, Poorter H, Schoen DJ (1996). Comparative ecology of seed size and dispersal [and Discussion]. Philos. Trans. Royal Soc. of London Ser. B: Biol. Sci.

[b800] Wolfe LM (1995). The genetics and ecology of seed size variation in a biennial plant, *Hydrophyllum appendiculatum* (Hydrophyllaceae). Oecologia.

[b63] Wu GL, Tian FP, Ren GH, Liu ZH (2011). Seed mass increase along altitude within four *Saussurea* (Asteraceae) species in Tibetan Plateau. Polish J. Ecol.

